# Emerging biomarkers of postoperative delirium at the intersection of neuroinflammation and neurodegeneration

**DOI:** 10.3389/fnagi.2025.1632947

**Published:** 2025-08-29

**Authors:** Kun Leng, Mervyn Maze, Odmara L. Barreto Chang

**Affiliations:** ^1^Department of Anesthesia and Perioperative Care, University of California, San Francisco, San Francisco, CA, United States; ^2^Department of Anesthesia and Perioperative Care, Center for Cerebrovascular Research, University of California, San Francisco, San Francisco, CA, United States

**Keywords:** postoperative delirium (POD), perioperative neurocognitive disorders (PND), neuroinflammation, Alzheimer’s disease, GFAP, IL-6 (interleukin 6), neurofilament light (NfL), tau

## Abstract

Postoperative delirium (POD) is a common and severe neuropsychiatric complication affecting older adults after surgery. POD is characterized by fluctuating cognitive disturbances, impaired attention, and altered consciousness, resulting in increased morbidity and mortality, prolonged hospital stays, and higher healthcare costs. Systemic inflammation induced by surgical trauma is implicated in the pathophysiology of POD, although the subsequent mechanisms that produce blood–brain barrier (BBB) dysfunction, neuroinflammation, and interactions with underlying dementia neuropathology have not been resolved. Recent advances in biomarker research have shed light on predictive and diagnostic tools for POD. Biomarkers linked to dementia neuropathology (e.g., hyperphosphorylated tau, amyloid beta), neuronal injury (e.g., total tau, neurofilament light chain), glial activation (e.g., glial fibrillary acidic protein), and systemic inflammation (e.g., interleukin-6) have shown promise. The feasibility of measuring the above biomarkers in easy-to-obtain biofluids such as blood is enhanced by technologies like single-molecule array immunoassays, enabling sensitive detection of central nervous system markers at femtomolar concentrations. Emerging evidence highlights associations between POD risk and these biomarkers, although findings often vary due to cohort heterogeneity and methodological differences. This review critically examines the existing literature on POD biomarkers, focusing on their relevance to dementia neuropathology, neuronal injury, neuroinflammation, and BBB integrity. While significant strides have been made, gaps in knowledge persist, emphasizing the need for larger, more standardized studies. Developing robust biomarkers could transform POD prediction, diagnosis, and management, ultimately improving outcomes for vulnerable surgical populations.

## 1 Introduction

Postoperative delirium (POD) is an acute neuropsychiatric syndrome characterized by fluctuating cognitive disturbances, impaired attention, altered levels of consciousness, and disrupted thinking that commonly affects older surgical patients, who are usually considered to be over 60 years of age. POD represents a significant clinical concern due to its association with poor postoperative patient outcomes (Gleason et al., 2015; Jin et al., 2020; Moskowitz et al., 2017). Despite its clinical importance, significant gaps in understanding the pathophysiological mechanisms underlying POD remain. The systemic inflammatory response induced by surgical trauma plays a major role (Saxena and Maze, 2018). Through poorly understood mechanisms, peripheral inflammatory factors cause endothelial dysfunction and increased permeability of the blood–brain barrier (BBB) (Devinney et al., 2023; Yang et al., 2017), facilitating infiltration of peripheral immune cells into the brain and subsequent neuroinflammation (Degos et al., 2013; Saxena et al., 2019; Subramaniyan and Terrando, 2019; Terrando et al., 2011; Vacas et al., 2013; Wang et al., 2020; Yang et al., 2020) and ultimately dysfunction of brain networks that underly cognition (Ditzel et al., 2023; Tanabe et al., 2020), resulting in delirium. Especially in elderly patients, there may also be complex interactions between POD pathophysiology and underlying dementia such as Alzheimer’s disease (Kunicki et al., 2023; Sun et al., 2024). Pre-existing cognitive impairment or dementia is known to increase the risk for POD (Dasgupta and Dumbrell, 2006; Sadeghirad et al., 2023), and the neuroinflammation caused by surgery may also accelerate underlying dementia (Kant et al., 2023; Lingehall et al., 2017).

Given the association of POD with increased morbidity, mortality, healthcare costs, and postoperative neurocognitive disorders, the search for biomarkers to improve the prediction, diagnosis, and management of POD is being actively pursued (Figueiredo and Devezas, 2024; Gonçalves et al., 2025; Marshall et al., 2025; Moazzen et al., 2024; O’Gara et al., 2021; Reekes et al., 2025). A biomarker is a “defined characteristic that is measured as an indicator of normal biological processes, pathogenic processes or responses to an exposure or intervention” (FDA-NIH Biomarker Working Group, 2016). In most cases, diagnostic biomarkers are analytes from biological specimens, usually biofluids, that can be measured to yield predictive information on the subsequent development of a pathological process. As we will discuss in this review, biomarkers of underlying dementia, neuronal injury, glial activation, and neuroinflammation have demonstrated significant associations with POD onset and severity in recent clinical studies (Table 1). Additionally, pre-clinical research highlights the relevance of endothelial dysfunction and BBB disruption in the development of POD, and clinical studies focusing on biomarkers of these pathophysiological mechanisms are emerging (Table 1).

**Table 1:** An overview of the literature on emerging biomarkers of POD pertaining to Figure 1.

Biomarker	Biofluid	Study	Surgery type (cohort)	Sample size	Associated with POD?
pTau217	Plasma	Liang et al. (2023)	Orthopedic	*n* = 139	Y
	Serum	McKay et al. (2022)	Cardiac	*n* = 38	N
pTau181	CSF	Liu et al. (2022)	Orthopedic (PNDABLE)	*n* = 1,471	Y
	CSF	Wang et al. (2022)	Orthopedic (PNDABLE)	*n* = 829	Y
	CSF	Cunningham et al. (2019)	Orthopedic	*n* = 282	N
	CSF	Chan et al. (2021)	Orthopedic	*n* = 199	N
	CSF	Witlox et al. (2011)	Orthopedic	*n* = 76	N
	CSF	Umoh et al. (2025)	Orthopedic	*n* = 158	N
	CSF	Idland et al. (2017)	Orthopedic (OOT)	*n* = 129	N
	CSF	Halaas et al. (2021)	Orthopedic (OOT)	*n* = 128	N
	CSF	Neerland et al. (2020)	Orthopedic (OOT)	*n* = 128	N
	CSF	Henjum et al. (2018)	Orthopedic (OOT)	*n* = 120	N
	CSF	Hov et al. (2017)	Orthopedic (OOT)	*n* = 98	N
	CSF	Wang et al. (2023)	Orthopedic	*n* = 138	Y
	CSF	Fong et al. (2021)	Orthopedic	*n* = 59	Y*
	CSF	Fong et al. (2024)	Orthopedic	*n* = 35 matched pairs	N
	CSF	Parker et al. (2022)	Vascular	*n* = 53	Y
Aβ	CSF	Wang et al. (2022)	Orthopedic (PNDABLE)	*n* = 829	Y
	CSF	Lin et al. (2023)	Orthopedic (PNDABLE)	*n* = 825	Y
	CSF	Lin et al. (2022)	Orthopedic (PNDABLE)	*n* = 740	Y
	CSF	Liu et al. (2022)	Orthopedic (PNDABLE)	*n* = 1,471	Y*
	CSF	Guo et al. (2024)	Orthopedic	*n* = 560	Y
	CSF	Cunningham et al. (2019)	Orthopedic	*n* = 282	Y
	CSF	Xie et al. (2014)	Orthopedic	*n* = 153	Y
	CSF	Wang et al. (2023)	Orthopedic	*n* = 138	Y
	CSF	Witlox et al. (2011)	Orthopedic	*n* = 76	Y
	CSF	Idland et al. (2017)	Orthopedic (OOT)	*n* = 129	Y
	CSF	Halaas et al. (2021)	Orthopedic (OOT)	*n* = 128	Y
	CSF	Neerland et al. (2020)	Orthopedic (OOT)	*n* = 128	Y
	CSF	Chan et al. (2021)	Orthopedic	*n* = 199	N
	CSF	Fong et al. (2024)	Orthopedic	*n* = 35 matched pairs	N
	CSF	Parker et al. (2022)	Vascular	*n* = 53	N
Total tau	CSF	Wang et al. (2022)	Orthopedic (PNDABLE)	*n* = 829	Y
	CSF	Lin et al. (2023)	Orthopedic (PNDABLE)	*n* = 825	Y
	CSF	Lin et al. (2022)	Orthopedic (PNDABLE)	*n* = 740	Y
	CSF	Liu et al. (2022)	orthopedic (PNDABLE)	*n* = 1,471	N
	CSF	Guo et al. (2024)	Orthopedic	n = 560	Y
	CSF	Parker et al. (2022)	Vascular	*n* = 53	Y
	CSF	Idland et al. (2017)	Orthopedic (OOT)	*n* = 129	Y
	CSF	Halaas et al. (2021)	Orthopedic (OOT)	*n* = 128	Y
	CSF	Neerland et al. (2020)	Orthopedic (OOT)	*n* = 128	Y
	CSF	Cunningham et al. (2019)	Orthopedic	*n* = 282	N
	CSF	Fong et al. (2020)	Mixed	*n* = 108	N
	CSF	Witlox et al. (2011)	Orthopedic	*n* = 76	N
	Plasma	Ballweg et al. (2021)	Mixed	*n* = 114	Y
	Urine	Baek et al. (2023)	Orthopedic	*n* = 91	Y
NfL	CSF	Halaas et al. (2018)	Orthopedic (OOT)	*n* = 314	Y
	CSF	Umoh et al. (2025)	Orthopedic	*n* = 158	Y
	CSF	Fong et al. (2020)	Mixed	*n* = 108	Y
	CSF	Parker et al. (2022)	Vascular	*N* = 53***	Y
	CSF	Zhou et al. (2022)	Neurosurgery	*n* = 40	Y
	CSF	Liu et al. (2023)	Mixed	*n* = 32	Y**
	Plasma	Ballweg et al. (2021)	Mixed	*n* = 114	N
	Urine	Baek et al. (2023)	Orthopedic	*n* = 91	N
GFAP	CSF	Fong et al. (2024)	Orthopedic	*n* = 35 matched pairs	Y*
	CSF	Cape et al. (2014)	Orthopedic	*n* = 43	N
	Urine	Baek et al. (2023)	Orthopedic	*n* = 91	N
	Plasma	Ballweg et al. (2021)	Mixed	*n* = 114	N
	Plasma	Liu et al. (2023)	Mixed	*n* = 32	Y
	Plasma	Fong et al. (2020)	Mixed	*n* = 108	Y*
	Plasma	Anderson et al. (2018)	Lung transplant	*n* = 155	N
	Plasma	Gailiušas et al. (2019)	Cardiac	*n* = 44	Y
CPAR	Plasma, CSF	Devinney et al. (2023)	Mixed non-cardiac	*n* = 207	Y
vWF, VCAM1	Plasma	Moazzen et al. (2024)	Mixed	*n* = 788	N
CCL2	Plasma	Kaźmierski et al. (2021)	Cardiac	*n* = 177	Y
	Plasma	Menzenbach et al. (2021)	Mixed	*n* = 118	Y

PNDABLE, Perioperative Neurocognitive Disorder And Biomarker Lifestyle. OOT, Oslo Orthogeriatrics Trial. *p value > 0.05 but trending towards statistical significance. **Emergence delirium. ***Excluding patients with spinal cord ischemia.

Ideally, biomarkers for POD should be sourced from easy-to-obtain biofluids such as blood or urine. Although cerebrospinal fluid (CSF) biomarkers provide direct insights into central nervous system (CNS) pathologies, the invasive nature of CSF sampling limits its clinical utility outside of research settings. Recent technological advances, such as single-molecule array (Simoa) immunoassays with exquisite detection sensitivity, have made it possible to reliably measure ultra-low levels of CNS-derived analytes in blood or urine (Dong et al., 2024), beyond what was achievable with traditional immunoassays. Consequently, peripherally circulating biomarkers for POD have gained attention due to their ease of collection, potential for repeated measurements over time, and utility.

**Figure 1:**
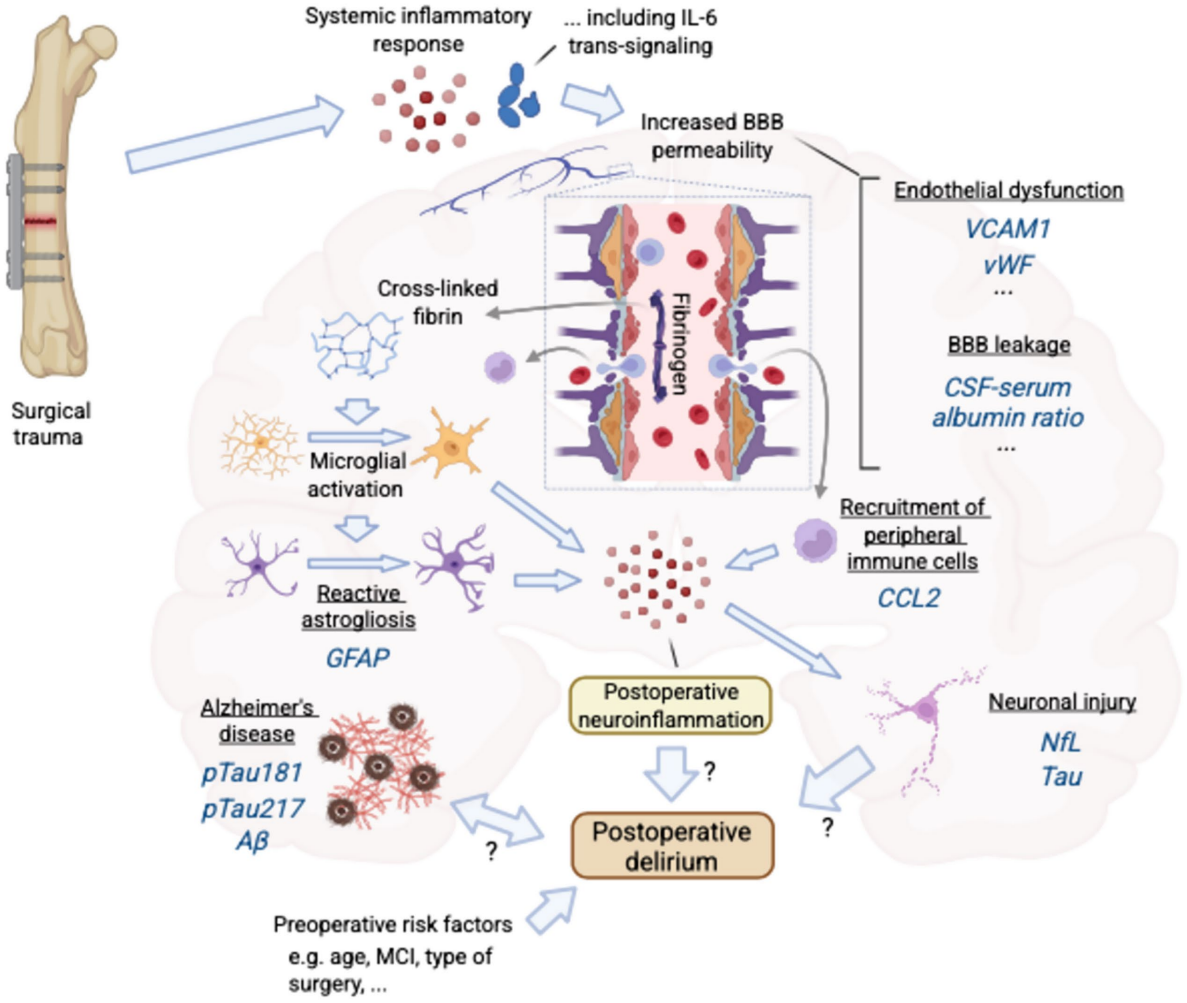
An overview of postoperative delirium pathophysiology and its potential biomarkers. MCI: mild cognitive impairment, BBB: blood–brain barrier.

This review critically evaluates the current evidence on biomarkers of POD in older adults, focusing on those pertaining to underlying dementia (specifically Alzheimer’s disease), neuronal injury, glial activation, neuroinflammation, endothelial dysfunction, and BBB integrity (Figure 1; Table 1). Furthermore, this review identifies current gaps in knowledge and proposes directions for future research aimed at enhancing our understanding and management of this complex postoperative complication.

## 2 Results

### 2.1 Alzheimer’s disease biomarkers: hyperphosphorylated tau (pTau), amyloid beta (Aβ)

Poor performance on preoperative cognitive assessments has been established as one of the strongest risk factors for the development of POD (Barreto Chang et al., 2022, 2023; Cao et al., 2019; Culley et al., 2017). Even the patient’s subjective experience of cognitive decline has some predictive value for POD (Namirembe et al., 2023), and subjective cognitive decline is itself associated with a number of negative outcomes, such as depression (Deiner et al., 2019; Zapater-Fajarí et al., 2024).

Although cognitive decline can occur for many reasons, the most common cause of mild cognitive impairment is Alzheimer’s disease, which is also the leading cause of dementia worldwide (Barreto Chang et al., 2023; Brookmeyer et al., 2007). Importantly, the neuropathological underpinnings of Alzheimer’s disease—the accumulation of amyloid beta (Aβ) deposits and hyperphosphorylated tau (pTau) tangles in the brain—occur well before the onset of clinical manifestations (Sperling et al., 2011), with pTau accumulation occurring earlier, possibly decades prior to clinical cognitive decline (Braak and Del Tredici, 2011). Increased concentration of Aβ or pTau in the CSF or blood reflects progression of Alzheimer’s neuropathology while the appearance of neurofilament light chain (NfL) indicates subsequent neuronal injury and loss (Jack et al., 2016). Recent advances in immunoassays for pTau have made it possible to reliably quantify pTau in the blood, with the newest blood pTau217 assays having excellent diagnostic performance superior to many blood pTau181 assays (Janelidze et al., 2023).

If the presence of Alzheimer’s disease neuropathology increases the risk for POD, it should be possible to risk stratify elderly patients for POD based on measurement of pTau or Aβ concentration in the blood or CSF. A number of studies have attempted to test this hypothesis, which we will discuss below, first focusing on pTau (pTau217 and pTau181), then on Aβ.

To our knowledge, only two cohort studies have examined pTau217 in surgical patients. Liang et al. (2023) conducted a study involving a cohort of 139 orthopedic (knee, hip and spine) surgical patients and demonstrated that elevated plasma levels of pTau181 and pTau217 were associated with fourfold and twofold increase in POD risk, respectively, which remained significant after controlling for age, education and preoperative cognition (Mini-mental State Examination); also, the severity of POD was associated with the preoperative levels of each of the blood-based pTau biomarkers. In a smaller cohort of cardiac surgery patients (*n* = 38), McKay et al. (2022) found significant postoperative elevations of both serum total tau and pTau (both pTau181 and pTau217), but only total tau was associated with POD.

As for pTau181, studies analyzing the large Perioperative Neurocognitive Disorder And Biomarker Lifestyle (PNDABLE) cohort (orthopedic surgery—hip or knee arthroplasty) in China have consistently found a significant positive association between preoperative CSF pTau181 levels and POD risk, for example in Liu et al. (2022) (*n* = 1,471) and Wang S. et al. (2022) (*n* = 829).

Inconsistent findings have been reported from smaller sized studies that have examined pTau181 in the CSF. Examining similar orthopedic surgery cohorts (hip or knee arthroplasty), Cunningham et al. (2019) (*n* = 282), Chan et al. (2021) (*n* = 199), Witlox et al. (2011) (*n* = 76), and Umoh et al. (2025) (*n* = 158) reported no significant association of preoperative CSF pTau181 with POD. Analyses of the Oslo Orthogeriatrics Trial cohort reported similar negative findings for preoperative CSF pTau181 as well [Idland et al., 2017 (*n* = 129), Halaas et al., 2021 (*n* = 128), Neerland et al., 2020 (*n* = 128), Henjum et al., 2018 (*n* = 120), Hov et al., 2017 (*n* = 98)]. However, Wang et al. (2023) (*n* = 138) did find a significant association in orthopedic patients with pTau181. The lack of consistency even extends to the same investigators: Fong et al. (2021) (*n* = 59) found a trend towards statistically significantly increased delirium incidence in orthopedic patients meeting amyloid, tau, and neurodegeneration (ATN) classification of AD, but in a separate case–control study (*n* = 35 matched pairs) failed to find a significant association of preoperative plasma or CSF pTau181 with POD (Fong et al., 2024). In a cohort of vascular surgery patients (thoracoabdominal aortic aneurysm repair), however, Parker et al. (2022) (*n* = 53) found that preoperative CSF pTau181 was associated with POD.

Given the consistent significant positive association between pTau181 with POD in the large PNDABLE studies, the lack of consistency among smaller sized studies may simply reflect lack of statistical power, although the heterogeneity of patient cohorts also needs to be considered, as the PNDABLE cohort is based in China whereas the other studies are based in the United States or Europe. The findings from Han Chinese patient cohorts may not generalize to predominantly Caucasian cohorts, and there may be environmental and sociocultural differences between China and Western countries that contribute as well. Overall, further studies in more diverse patient cohorts are needed to establish whether preoperative pTau is useful for POD risk prediction, especially using the newer, more-sensitive, blood-based assays measuring pTau217, which have superior diagnostic performance.

With respect to Aβ, analyses of the PNDABLE cohort for the most part found a significant association of lower CSF Aβ_42_ with POD [Wang et al., 2022 (*n* = 829), Lin et al., 2023 (*n* = 825), Lin et al., 2022 (*n* = 740)], although the *p* value in Liu et al. (2022) (*n* = 1,471) was borderline (*p* = 0.06). In other orthopedic cohorts, Guo et al. (2024) (*n* = 560), Cunningham et al. (2019) (*n* = 282), Xie et al. (2014) (*n* = 153), Wang et al. (2023) (*n* = 138), Witlox et al. (2011) (*n* = 76), and analyses of the Oslo Orthogeriatrics Trial [Idland et al., 2017 (*n* = 129), Halaas et al., 2021 (*n* = 128), Neerland et al., 2020 (*n* = 128)] have consistently reported similar findings. However, there were several studies which reported no significant association: Chan et al. (2021) (*n* = 199, orthopedic), Fong et al. (2024) (*n* = 53 matched pairs, orthopedic), and Parker et al. (2022) (*n* = 53, vascular).

Overall, the preponderance of evidence favors lower CSF Aβ_42_ as predictive of increased risk for POD, in agreement with lower CSF Aβ_42_ as a biomarker of Alzheimer’s disease. Again, the inconsistency among the smaller-sized studies may be due to lack of statistical power; differences in the assays used to detect Aβ_42_ may have also contributed.

### 2.2 Markers of neuronal injury: total tau, NfL

While pTau is specific for Alzheimer’s disease, total tau in CSF or blood more likely reflects non-specific neuronal injury, as tau is an abundant microtubule binding protein in neurons. Similarly, neurofilament light chain (NfL) is a neuron-specific cytoskeleton protein that is elevated in the CSF or blood in the setting of neuronal injury. With respect to POD, pre-existing neuronal injury (e.g., from underlying Alzheimer’s disease) would likely increase the risk for POD, and certain types of surgery may induce neuronal injury (e.g., microscopic emboli or microcirculatory dysfunction in the brain from cardiopulmonary bypass), which could further increase the risk for POD and long-term cognitive impairment. Interestingly, in patients who have undergone cardiac surgery, the presence of POD increases the risk for developing subsequent dementia within 5 years (Davis et al., 2012; Goldberg et al., 2020; Lingehall et al., 2017).

A number of studies have examined neuronal injury markers such as total tau or NfL in the blood or CSF in surgical cohorts. For total tau, most studies examined CSF collected preoperatively and reported increased tau as a predictor of POD. Analyses of the PNDABLE cohort for the most part have demonstrated increased preoperative CSF tau as a risk factor, with Wang S. et al. (2022) (*n* = 829), Lin et al. (2023) (*n* = 825), and Lin et al. (2022) (*n* = 740) reporting a significant association, whereas Liu et al. (2022) (*n* = 1,471) did not. In other orthopedic cohorts, Guo et al. (2024) (*n* = 560), Parker et al. (2022) (*n* = 53), and analyses of the Oslo Orthogeriatrics Trial (Idland et al., 2017 (*n* = 129), Halaas et al., 2021 (*n* = 128), Neerland et al., 2020 (*n* = 128)) also reported an association. However, Cunningham et al. (2019) (*n* = 282, orthopedic), Fong et al. (2020) (*n* = 108, mixed surgical cases), and Witlox et al. (2011) (*n* = 76, orthopedic) did not find a statistically significant association.

There are only a few studies that have examined total tau in other biofluids besides CSF as a potential non-invasive biomarker. Ballweg et al. (2021) (*n* = 114, mixed surgical cases) measured total tau in plasma at multiple time points and reported that the change in plasma total tau was greater in patients with POD and correlated with delirium severity. Baek et al. (2023) (*n* = 91, orthopedic) measured total tau in urine extracellular vesicles (from urine collected postoperatively) and found that total tau was higher in patients who developed POD vs. those who did not.

As for NfL, the majority of studies have reported increased blood or CSF NfL as a risk factor for POD. Halaas et al. (2018) (*n* = 314, orthopedic), Umoh et al. (2025) (n = 158, orthopedic), Fong et al. (2020) (*n* = 108, mixed surgical cases), Parker et al. (2022) (vascular, excluding patients with putative spinal cord ischemia), and Zhou et al. (2022) (*n* = 40, DBS implant neurosurgery in patients with Parkinson’s disease) reported that increased CSF NfL preoperatively was associated with POD. Liu et al. (2023) (*n* = 32, mixed surgical cases) reported an association with emergence delirium. In contrast, Ballweg et al. (2021) (*n* = 114, mixed surgical cases) did not find an association between POD and plasma NfL, and Baek et al. (2023) (*n* = 91, orthopedic) did not find differences in urine extracellular vesicle NfL in patients with POD vs. those without.

Overall, the preponderance of evidence favors markers of neuronal injury being predictive of POD risk, although it remains to be determined whether total tau or NfL is superior. Small sample sizes and differences in the patient demographics of the clinical cohorts and the immunoassays used likely contribute to the discrepancy of study findings.

It should be noted that other markers have been used as surrogates of neuronal injury. For example, ubiquitin carboxyl-terminal hydrolase L1 (UCH-L1) has been used in studies on traumatic brain injury (Blyth et al., 2011; Brophy et al., 2011) and has been repurposed as a possible biomarker for POD, for example, in Lopez et al., where higher postoperative levels of UCH-L1 was associated with increased POD risk (Lopez et al., 2020).

### 2.3 Markers of reactive astrocytes: glial fibrillary acidic protein (GFAP)

In many contexts, neuroinflammation causes reactive astrogliosis, a hallmark of which is increased expression of GFAP, a protein specific to astrocytes in the adult brain. Increased expression of GFAP in the CNS would then lead to increased concentration of GFAP in the CSF and also blood, presumably through increased permeability of the blood–brain barrier in the setting of neuroinflammation. For example, patients with severe COVID-19 have elevated plasma GFAP (Cooper et al., 2020; Sahin et al., 2022), which was associated with subsequent mild cognitive impairment (Bark et al., 2023), and blood GFAP is also becoming recognized as a biomarker for Alzheimer’s disease (Ally et al., 2023; Chatterjee et al., 2021; Pereira et al., 2021; Sánchez-Juan et al., 2024). Thus, given that neuroinflammation is a well-established part of POD pathophysiology, it would be reasonable to hypothesize that there would be postoperative reactive astrogliosis and hence elevated concentration of GFAP in the CSF or blood. Indeed, reactive astrogliosis is seen in mouse models of POD (Hua et al., 2023), and reactive astrogliosis has been reported in post-mortem samples from patients with delirium (van Munster et al., 2011), although it was unclear whether the cases reflected medical delirium or POD. Alternatively, pre-existing reactive astrogliosis, for example in reaction to underlying Alzheimer’s neuropathology, may also increase the risk for POD.

A number of studies have examined CSF or blood GFAP in POD. However, studies have reported conflicting findings regarding the association between GFAP and POD. For example, examining orthopedic patients, Fong et al. (2024) (*n* = 35 matched pairs, Simoa assay) found that both preoperative plasma and CSF GFAP were nearly twofold higher in patients with POD and was associated with increased POD risk, but the results only trended towards statistical significance. In a separate orthopedic cohort, Cape et al. (2014) (*n* = 43, traditional immunoassay) did not find an association between CSF GFAP and POD. Baek et al. (2023) (*n* = 91, Simoa assay) examined GFAP in postoperative urine samples from orthopedic patients and did not find an association with POD.

With respect to mixed surgical cohorts, Ballweg et al. (2021) (*n* = 114, Simoa assay) did not find any association between plasma GFAP and POD, whereas Liu et al. (2023) (n = 32, traditional immunoassay) found that the increase in plasma GFAP postoperatively compared to preoperatively was associated with increased POD risk, and Fong et al. (2020) (*n* = 108, Simoa assay) reported a trend towards statistically significant association of preoperative and postoperative plasma GFAP with POD risk.

As for surgeries involving cardiopulmonary bypass, Anderson et al. (2018) (*n* = 155, traditional immunoassay) examined GFAP in lung transplant patients and did not find an association between postoperative plasma GFAP and POD, but GFAP was not consistently detected, presumably due to the limits of the traditional GFAP immunoassay used in that study. Gailiušas et al. (2019) (*n* = 44, traditional immunoassay) found a significant increase in plasma GFAP after surgery as well as higher plasma GFAP in patients with POD in cardiac surgery patients.

Overall, the evidence for the usefulness of GFAP as a biomarker for POD is mixed, likely due to the small number of studies, the heterogeneity of surgical cohorts examined, and heterogeneity in the GFAP assays employed. Further investigation of GFAP in larger cohorts using Simoa-based assays or assays with equal sensitivity is warranted.

### 2.4 Systemic and neuro-inflammation: the role of IL-6

IL-6 is a key mediator of the neuroinflammatory cascade following aseptic surgical trauma. Tissue injury releases damage-associated molecular patterns (e.g., HMGB1), which activate pattern recognition receptors such as RAGE on bone marrow-derived monocytes (BM-DMs), leading to NF-κB-mediated upregulation of inflammatory cytokines, including IL-6 (Cibelli et al., 2010). This systemic inflammatory response can disrupt the blood–brain barrier (BBB), allowing peripheral cytokines and immune cells to infiltrate the CNS (Degos et al., 2013; Hu et al., 2018). Activated microglia and recruited BM-DMs in the hippocampus are capable of releasing IL-6, which may disrupt synaptic plasticity and thereby impair memory (Hu et al., 2018).

IL-6 operates via two distinct pathways: classic signaling through membrane-bound IL-6Rα and trans-signaling via soluble IL-6R (sIL-6R). The latter forms an IL-6/sIL-6R complex that interacts with gp130 on cells lacking IL-6Rα, thereby expanding the range of IL-6’s proinflammatory effects (Barreto Chang and Maze, 2022; Rose-John, 2021). Recent preclinical studies demonstrate that IL-6 trans-signaling in hippocampal CA1 neurons is both necessary and sufficient to induce postoperative cognitive impairment (Hu et al., 2022). Post-surgical increases in hippocampal IL-6 and CSF sIL-6R correlate with memory deficits and Stat3 phosphorylation (Hu et al., 2022). Pharmacological inhibition of trans-signaling with sgp130Fc prevents these deficits (Hu et al., 2022), confirming the causal role of IL-6 trans-signaling in POD pathogenesis.

Clinical meta-analyses show that elevated pre- and postoperative IL-6 levels are associated with increased POD risk (Liu et al., 2018; Noah et al., 2021). Advanced age and preexisting cognitive impairment, major risk factors for POD, are linked to chronic low-grade inflammation (“inflammaging”), which includes elevated IL-6 levels (Ferrucci et al., 1999; Singh-Manoux et al., 2014; Wichmann et al., 2014). Aged mice show increased hippocampal IL-6 and sIL-6R and heightened microglial sensitivity to IL-6 signaling (Burton et al., 2013; Garner et al., 2018; Porcher et al., 2021). Interestingly, changes in systemic IL-6 levels over the course of the first postoperative year are correlated with changes in executive function in patients (Taylor et al., 2023).

### 2.5 Markers of endothelial and blood brain barrier (BBB) dysfunction

Endothelial dysfunction and compromised blood–brain barrier (BBB) integrity are increasingly recognized as crucial components in the pathophysiology of postoperative delirium. Preclinical investigations of POD using mouse models have demonstrated increased BBB permeability and ensuing peripheral immune cell infiltration into the brain parenchyma after orthopedic surgery (Terrando et al., 2011; Yang et al., 2019). However, there are currently relatively few clinical studies examining markers of endothelial and BBB dysfunction in POD.

Elevated CSF-to-plasma albumin ratio (CPAR) is considered a marker of increased BBB permeability, because albumin is normally not abundant in CSF compared to plasma. Taylor et al. (2022) (*n* = 25, mixed surgical cases) reported that surgery induced an increase in CPAR and also higher CSF levels of S100β, which is also considered a marker of increased BBB permeability. Payne et al. (2024) (*n* = 24, aortic aneurysm repair) further supported these findings by showing postoperative elevation in CPAR, and also found that surgery increased CSF fibrinogen, a marker closely linked to BBB integrity impairment and neuroinflammation (Ryu et al., 2009). Importantly, in a larger cohort, Devinney et al. (2023) (*n* = 207, mixed non-cardiac surgical cases) found that a higher CPAR was associated with increased incidence of POD after adjusting for clinical covariates. Thisayakorn et al. (2024) (*n* = 59) reported elevated plasma IgG/IgA antibodies against tight junction proteins (zonulin, occludin, claudin), which are integral to BBB integrity, in patients with POD.

Although the above studies supported *post*operative BBB dysfunction, Moazzen et al. (2024) (*n* = 788, mixed surgical cases) examined *pre*operative plasma levels of markers of endothelial dysfunction (including asymmetric and symmetric dimethylarginine, ICAM-1, VCAM-1, vWF) and did not find an association with POD risk, suggesting that the link between endothelial and BBB dysfunction with POD may be dynamic. For example, baseline *preo*perative levels of endothelial dysfunction markers alone may not be predictive of POD, whereas the preoperative to postoperative change may be predictive.

Interestingly, a number of studies have associated proteins involved in immune cell recruitment, e.g., chemokines and cell adhesion molecules, with POD. For example, both preoperative (Kaźmierski et al., 2021) (*n* = 177, cardiac) and postoperative (Menzenbach et al., 2021) (*n* = 118, mixed surgical cases) blood CCL2 (chemokine) levels are positively associated with POD risk. Mietani et al. (2019) (*n* = 117, mixed surgical cases) found that postoperative plasma P-selectin (endothelial adhesion molecule for immune cells) levels were associated with elevated plasma concentration of phosphorylated neurofilament heavy chain (pNfH), which was used as a biomarker of POD (sensitivity of 56% and specificity of 90%). The results above lend support to the preclinical evidence demonstrating a role for peripheral immune cell infiltration into the brain in the pathophysiology of POD (D’Mello et al., 2009; Degos et al., 2013; Xu et al., 2017), and preliminary clinical studies have corroborated this as well (Berger et al., 2019).

Overall, further investigation of markers of endothelial and BBB dysfunction in POD is needed, especially studies that follow these markers both preoperatively and postoperatively. Correlating the markers with the cognitive trajectories of these patients before and after surgery will yield insights into how different patient intrinsic characteristics and the immune response to surgical trauma may contribute to different outcomes.

## 3 Discussion

### 3.1 Limitations of our mini-review

Our mini-review aims to bring increased attention to emerging biomarkers of POD pertaining to our understanding of its pathophysiology. Although a systematic review and meta-analysis of the existing literature on biomarkers of POD [e.g. (Lozano-Vicario et al., 2023)] would certainly benefit the field of POD research, we feel that such an endeavor is premature for many of the topics discussed here apart from IL-6 and AD biomarkers, for which there are several excellent meta-analyses available (Geng et al., 2024; Wang S. et al., 2022).

### 3.2 Future directions

Several crucial avenues remain to be explored in future research to deepen our understanding of postoperative delirium and enhance its management.

First, elucidating the detailed molecular and cellular mechanisms linking pro-inflammatory cytokines, particularly IL-6, to neuronal injury and subsequent elevation of neurodegenerative biomarkers such as tau, pTau, and NfL is essential. Clarifying these pathways could significantly advance our understanding of POD pathophysiology, identify other possible biomarkers, and guide targeted anti-inflammatory therapeutic strategies.

Second, further investigation into how endothelial dysfunction and increased blood–brain barrier (BBB) permeability contributes to POD initiation and progression is warranted. Detailed characterization of the vascular changes and molecular pathways involved in BBB disruption (see Figure 1) could inform targeted interventions aimed at preserving endothelial integrity and mitigating delirium risk.

Finally, longitudinal studies tracking biomarker changes preoperatively, perioperatively, and postoperatively are necessary to better understand temporal biomarker dynamics and their predictive accuracy for POD. With further investigations of the biomarkers discussed in this review and larger clinical cohorts, we envision that a multi-modal POD prediction score integrating baseline patient demographics, preoperative cognitive evaluation, clinical metadata, and plasma biomarkers is a tool that could feasibly be developed in the near future, potentially with the aid of machine learning or artificial intelligence, for early identification of patients at risk.
